# Daratumumab monotherapy for refractory lupus nephritis

**DOI:** 10.1038/s41591-023-02479-1

**Published:** 2023-08-10

**Authors:** Dario Roccatello, Roberta Fenoglio, Ilaria Caniggia, Joelle Kamgaing, Carla Naretto, Irene Cecchi, Elena Rubini, Daniela Rossi, Emanuele De Simone, Giulio Del Vecchio, Martina Cozzi, Savino Sciascia

**Affiliations:** University Center of Excellence on Nephrological, Rheumatological and Rare Diseases (ERK-net, ERN-Reconnect and RITA-ERN Member) including Nephrology and Dialysis Unit and Center of Immuno-Rheumatology and Rare Diseases (CMID), Coordinating Center of the Interregional Network for Rare Diseases of Piedmont and Aosta Valley (North-West Italy), San Giovanni Bosco Hub Hospital, ASL Città di Torino and Department of Clinical and Biological Sciences of the University of Turin, Turin, Italy

**Keywords:** Lupus nephritis, Lupus nephritis

## Abstract

Treatment-refractory lupus nephritis (LN) has a high risk of a poor outcome and is often life-threatening. Here we report a case series of six patients (one male and five females) with a median age of 41.3 years (range, 20–61 years) with refractory LN who received renal biopsies and were subsequently treated with intravenous daratumumab, an anti-CD38 monoclonal antibody (weekly for 8 weeks, followed by eight biweekly infusions and up to eight monthly infusions). One patient did not show any improvement after 6 months of therapy, and daratumumab was discontinued. In five patients, the mean disease activity, as assessed by the Systemic Lupus Erythematosus Disease Activity 2000 index, decreased from 10.8 before treatment to 3.6 at 12 months after treatment. Mean proteinuria (5.6 g per 24 h to 0.8 g per 24 h) and mean serum creatinine (2.3 mg dl^−1^ to 1.5 mg dl^−1^) also decreased after 12 months. Improvement of clinical symptoms was accompanied by seroconversion of anti-double-stranded DNA antibodies; decreases in median interferon-gamma levels, B cell maturation antigen and soluble CD163 levels; and increases in C4 and interleukin-10 levels. These data suggest that daratumumab monotherapy warrants further exploration as a potential treatment for refractory LN.

## Abstract (web summary)

In a case series of six patients with refractory lupus nephritis who were treated off-label with an anti-CD38 monoclonal antibody, five patients achieved complete or partial renal responses by 12 months of follow-up.

## Main

Lupus nephritis (LN) occurs in 35–45% of patients with systemic lupus erythematosus (SLE) and still represents a challenge to clinicians^1^.

Both the European League Against Rheumatism (EULAR)/European Renal Association (ERA)–European Dialysis and Transplant Association (EDTA) and Kidney Disease: Improving Global Outcomes (KDIGO) recommendations indicate either mycophenolate mofetil (MMF) or cyclophosphamide (CYC) given intravenously twice a month for 3 months in combination with three pulses of methylprednisolone, followed by slowly tapering oral prednisone, to be the standard of care (SOC) for remission induction. However, there is a consistent minority of patients who do not achieve complete renal response and are classified as refractory.

Refractory LN is commonly defined as either no response to SOC—that is, failure to improve within 3–4 months or not achieving partial response after 6–12 months—or complete response to SOC after 2 years of treatment^2^. A more comprehensive definition should include the double attempt with both MMF-based and CYC-based regimens^2^. However, concerns arise on the possible adverse effects of a prolonged ineffective immunosuppression, even though LN must require a relatively long time to achieve response, especially in patients with nephrotic-range proteinuria at baseline who often need up to 12 more months to achieve complete clinical response.

The equivalence between MMF-based and CYC-based regimens as SOC is still debated. Some evidence suggests differing efficacies of the MMF-based regimen and the CYC-based regimen in different ancestries^3,4^. Moreover, the equivalence of the EULAR scheme^3^ and the National Institutes of Health protocol^4^ is still a controversial topic. Low-dose MMF + tacrolimus has also been shown to be as effective as intravenous CYC in induction and maintenance therapy in a Chinese cohort^5^. Ongoing studies will clarify the role of the multi-target therapy in ethnically diverse populations.

Developing specific treatment strategies for refractory LN is important to limit the risks of poor outcome, and several potential alternative options have been under investigation (proteasome inhibitors, plasma exchange and stem cell transplantation^6–9^).

In a recent randomized controlled trial, the anti-BLyS monoclonal antibody belimumab was evaluated as an add-on-therapy^10^. This prompted consideration of the use of belimumab in combination with SOC in the management of difficult cases of LN^11^, albeit the effects of belimumab in heavy proteinuria seem to be not impressive.

Voclosporin is a new generation calcineurin inhibitor that decreases interleukin-2, interferon-gamma (INF-γ) and tumor necrosis factor-alpha (TNF-α) production by T cells. In the AURORA trial^12^, patients given voclosporin on top of steroids and MMF achieved a significantly higher rate of complete renal response compared to patients receiving placebo plus MMF-based SOC.

In the TULIP-LN trial^13^, anifrolumab, a monoclonal antibody directed at interferon-alpha (INF-α) and beta receptor subunit 1, given at greater dose than that approved by the US Food & Drug Administration (FDA) for the treatment of non-renal SLE, was found to achieve more complete renal response than placebo when added to MMF-based SOC. A phase 3 trial is ongoing. In patients with LN treated with anti-CD20 depleting agents, a considerable variability in peripheral blood B cell depletion can be observed. Reaching complete peripheral depletion, together with a prolonged duration of complete peripheral depletion (>71 d), have been associated with complete response^14^. This goal has been obtained by a short-term intensified B cell depletion therapy (IBCDT) protocol^2,15^ consisting of a combination therapy of rituximab (RTX) (four weekly doses of 375 mg m^−2^, followed by two further doses after 1 month and 2 months) and CYC given at sub-immunosuppressive doses (two pulses 10 mg kg^−1^ reduced by 30% in patients with impaired renal function) aimed at potentiating the B-cell-depleting effects of RTX. This combined regimen proved to be highly effective even in the long term (5.5 years, range, 3.7–7 years) without immunosuppressive maintenance therapies^2,15–17^. A prolonged B cell depletion can also be achieved by the type II monoclonal antibody anti-CD20 obinutuzumab, which reduces CD20 internalization and does not elicit CD20 redistribution^18^.

An approach that selectively depletes plasma cells secreting the pathogenic antibodies would be a substantial advance in the SLE setting^2^. CD38 is highly expressed on the surface of many immune cells, especially plasma cells (PCs), and a cell-type-specific dysregulation of CD38 expression has been observed in patients with SLE. Daratumumab, which is a recently emerged monoclonal antibody directed at CD38, has been shown to deplete highly expressing CD38 PCs in the bone marrow. Data regarding the efficacy of daratumumab in SLE are presently limited to two patients with refractory disease who were reported to be successfully treated with this agent in addition to receiving continued immunosuppression and complemented by maintenance therapy with the antibody belimumab^19^.

In this case series, we evaluated the efficacy and safety of daratumumab given without any other immunosuppressant or agents targeting B-cell-activating factor in a discrete cohort of patients with refractory LN, a still unexplored setting of patients, in whom SOC and rescue treatments had failed.

## Results

**Fig. 1:**
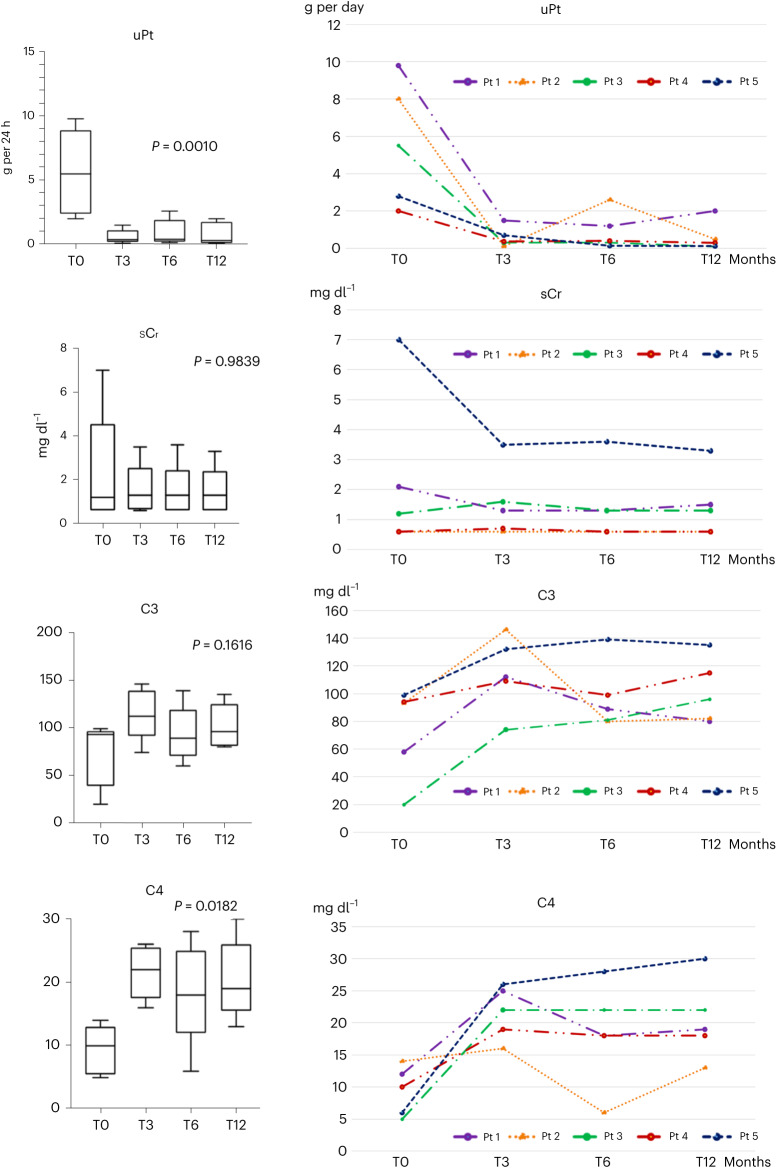
Laboratory profiles. Trend of proteinuria, sCr, C3 and C4 in responder patients at baseline and at 3 months, 6 months and 12 months. *n* = 5 biologically independent samples were used for each determination (one per patient/timepoint). Box plots show median and 1st–3rd interquartile range; whiskers indicate minimum–maximum. *P* ≤ 0.05 was considered significant. uPt, proteinuria.

We present six cases of refractory LN (one male and five females), median age 41.3 years (range, 20–61 years), treated with daratumumab monotherapy as a rescue therapy. One patient did not show clinical response after 6 months of therapy, and daratumumab was discontinued. Five patients continued to be treated, reaching a 12-month observation with 22 infusions. Renal biopsy performed before daratumumab starting revealed a class IV LN in one patient, a class V LN in one patient, a class III + V LN in one patient and a class IV + V LN in the other two. At the time of treatment beginning, the mean serum creatinine (sCr) value was 2.3 mg dl^−1^ (range, 0.6–7 mg dl^−1^); the mean estimated glomerular filtration rate (eGFR) was 62 ml/min/1.73 m^2^ (range, 9–111 ml/min/1.73 m^2^); and the mean proteinuria was 5.6 g per 24 h (range, 2–9.8 g per 24 h). At 3 months, all of these five patients achieved an overall response—specifically, three patients achieved a complete renal response, and the other two achieved a partial renal response. A significant decrease in proteinuria from 5.6 g per 24 h to 0.59 g per 24 h, 0.9 g per 24 h and 0.8 g per 24 h (*P* = 0.0010) at 3 months, 6 months and 12 months, respectively, was observed (Fig. 1). The mean value of sCr decreased from 2.3 mg dl^−1^ to 1.5 mg dl^−1^ (*P* = 0.98) at 12 months (Fig. 1). Of note, two patients experienced a mild rise in sCr (in one case transient); two patients experienced a decrease in sCr; and in one patient, no change was observed. C3 and C4 levels increased from 72.8 (range, 20–99) to 101.6 (range, 78.0–135) mg dl^−1^ (*P* = 0.16) (Fig. 1) and from 9.4 (range, 5–14) to 20.4 (range, 13–30) mg dl^−1^ (*P* = 0.0182), respectively (Fig. 1). A decrease in immunoglobulin G (IgG) mean levels from 971.7 mg dl^−1^ (range, 251–1546) to 512.3 mg dl^−1^ (range, 88–957), 481.7 (range, 84–878) mg dl^−1^ and 508.4 mg dl^−1^ (range, 192–685) at 3 months, 6 months and 12 months, respectively, was observed.

**Fig. 2:**
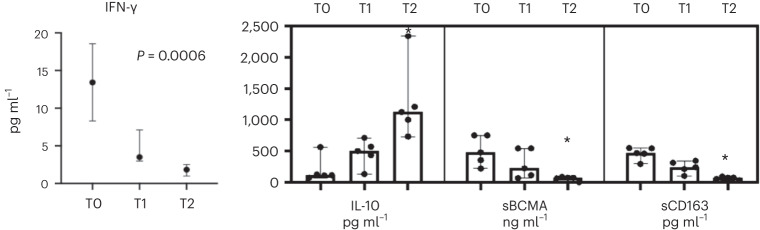
Serum concentrations of the INF-γ (shown as median and interquartile range), IL-10, sBCMA and sCD163 and scatter dot plots with results shown as median and range. *P < 0.05. TO, baseline; T6, 6 months; T12, 12 months. *n* = 5 biologically independent samples were used for each determination (one per patient/timepoint). The Wilcoxon matched‐pairs signed‐rank test was applied to investigate variations in parameters after treatment. *P* ≤ 0.05 was considered significant.

The median INF-γ values decreased from 13.43 pg ml^−1^ to 1.84 pg ml^−1^ (*P* = 0.0006). Interleukin (IL)-10 level increased from 113.56 pg ml^−1^ to 1,123.50 pg ml^−1^ (*P* = 0.0005). Serum B cell maturation antigen (sBCMA) and soluble CD163 decreased from 451.34 ng ml^−1^ to 64.45 ng ml^−1^ (*P* = 0.0005) and from 467.20 pg ml^−1^ to 71.45 pg ml^−1^ (*P* = 0.045), respectively (Fig. 2).

Patients were monitored for percentage of CD38^+^ cells in the peripheral blood, and CD38^+^ cells were undetectable for the time of treatment.

The Systemic Lupus Erythematosus Disease Activity 2000 (SLEDAI-2K) index decreased from 10.8 at baseline to 5.6 and 3.6 at 6 months and 12 months, respectively (*P* = 0.03).

**Fig. 3:**
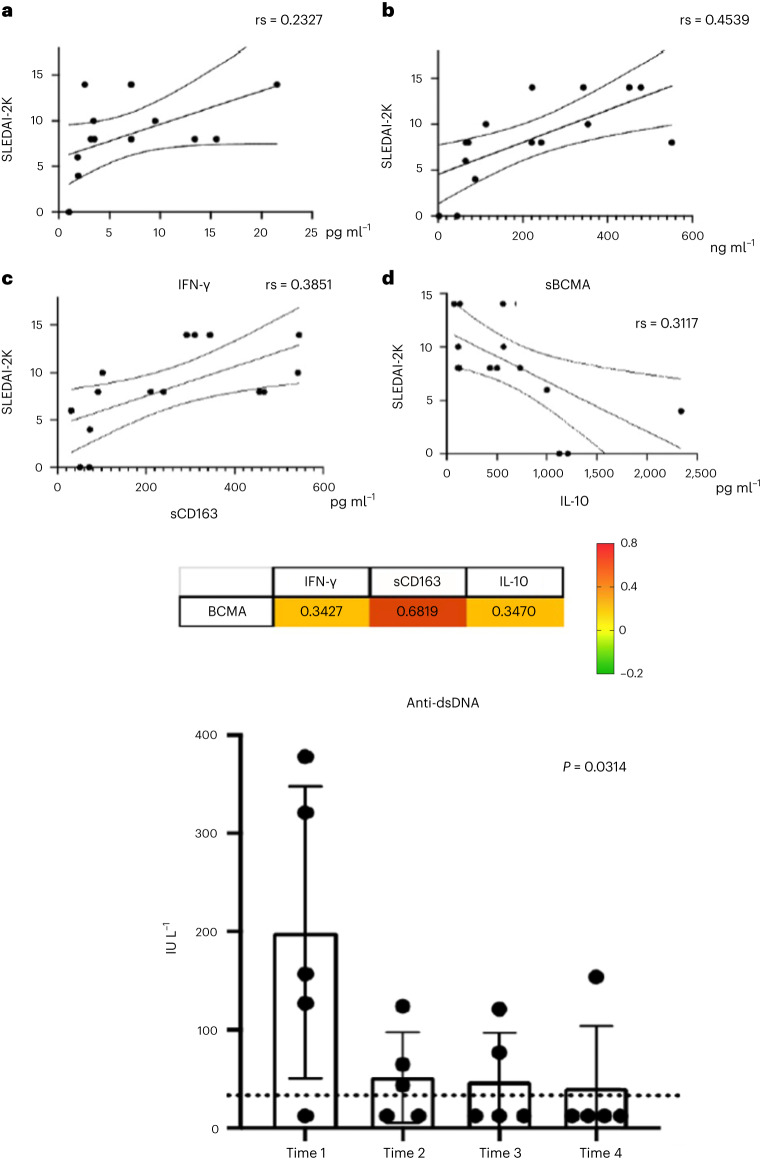
Immunological profiles. **a**–**d**, Top panels: Associations of serum. IFN-γ (pg ml^−1^) (**a**), sBCMA (ng ml^−1^) (**b**), sCD163 (pg ml^−1^) (**c**), IL-10 (pg ml^−1^) (**d**), and disease activity in SLE patients. Cytochines levels are expressed in ng ml^−1^. Rs: Spearman’s correlation coefficient, SLEDAI-2K: Systemic Lupus Erythematosus Disease Activity 2000 Index. Middle panel: Correlation matrix heat map of sBCMA and the cytokines IFN-γ, sCD163 and IL-10. Bottom panel: Serum levels of anti-dsDNA (scatter dot plots with results shown as median and range). T0, baseline; T3, 3 months; T6, 6 months; T12, 12 months. The Spearman rank correlation coefficient (rs) was used to assess correlations between parameters. *P* ≤ 0.05 was considered significant.

As shown in Fig. 1c, sBCMA had a good correlation (Spearman correlation coefficient (rs) = 0.4539) with disease activity, whereas sCD163 (rs = 0.3851), IL-10 (rs = 0.3117) and IFN-γ (rs = 0.2327) showed a lower correlation with SLEDAI-2K. The correlation between sBCMA and IFN-γ, sCD163 and IL-10 is shown in Fig. 3.

Anti-DNA antibodies decreased from 157 IU L^−1^ to 12 IU L^−1^ (*P* = 0.03) (Fig. 1e). Two patients experienced an asymptomatic severe acute respiratory syndrome coronavirus 2 (SARS-Co-2) infection (patient (Pt) 1 after the 12th administration and Pt 4 after the 10th administration) without effects on renal response. In one of them (Pt 4), after infection, anti-double-stranded DNA (dsDNA) antibodies turned to positive. In the other one (Pt 1), a temporary increase of antibody anti-dsDNA titer and proteinuria level was observed after coronavirus disease 2019 (COVID-19) vaccination (after the 17th administration).

All five patients had a persistent response at the last visit. Pt 2, who achieved a complete renal response at 3 months, needed a temporary treatment discontinuation due to a miscarriage with subsequent new flare of the SLE. After restarting daratumumab, the patient achieved a partial renal response with a progressive decrease in proteinuria level (from 5.3 g per 24 h to 0.8 g per 24 h at the 18th administration) and persistent normal renal function.

No serious adverse event (that is, requiring hospitalization) was recorded. One patient (Pt 4) reported mild redness of the face associated with a warm sensation during the first infusion. The symptoms resolved spontaneously without the need for specific treatments.

At 12 months, the residual dose of prednisone in these patients was 5 mg per day.

## Discussion

The main challenges in the treatment of severe LN are achieving a prompt response within months, maintaining this response and avoiding flares, preventing renal impairment in the long term, avoiding damage and comorbidities and fulfilling these objectives assuring a maximal quality of life.

Patients for whom the first-line treatment with SOC (MMF and CYC/azathioprine) failed or resulted in an inadequate response are considered as having a refractory disease^2^. This condition is associated with poor outcome, including end-stage renal disease^1^. This is why it is of upmost importance to develop specific treatment strategies for these patients^2^.

No agreement exists on the definition of ‘refractory LN’. Refractoriness should be fully determined by persistent histologic signs of activity despite putatively effective therapy. However, there is a latency between achieving clinical remission and substantial changes of histologic features. For this reason, refractory LN continues to be defined on a clinical ground. Several factors may contribute^20^, such as poor medication adherence, poor tolerability of therapy and adverse events and delayed patient referral causing irreversible injuries prevailing in the clinical picture. Geographic, ethnic, genetic and other epidemiological factors can also influence treatment response. However, the choice of the immunosuppressive treatment plays an important role. For instance, protocols based on higher-dose oral glucocorticoids have better response rates but at the price of higher toxicity^21^.

Additionally, none of currently available treatments, including standard MMF-based and CYC-based regimens, multi-target and add-on therapies (anti-BLyS, anti-INF, old and novel anti-CD20 monoclonal antibodies and voclosporin) and rescue therapies, including proteasome inhibitors, plasma exchange and stem cell transplantation, is able to assure long-lasting complete response in the totality of patients, and the issue of refractoriness to both conventional and rescue therapy, albeit limited to a restricted number of cases, remains a challenge for clinicians.

A growing body of evidence is supporting the rationale of an anti-CD38 strategy in these cases. CD38 is a glycoprotein expressed on the surface of many leucocytes participating in different cellular activities, including signal transduction, cell adhesion and calcium signaling^22^. Patients with SLE have been observed to present with abnormalities of CD38 expression when analyzed at cell-type-specific level^23^. Plasma cells express CD38, and the monoclonal antibody daratumumab was found to be effective in depleting bone marrow CD38^+^ PCs.

The use of daratumumab combined with belimumab in addition to continued standard immunosuppression has been recently explored in two patients with refractory SLE^19^ with promising results.

In the present study, six patients with refractory LN treated with daratumumab monotherapy are reported. They include one male and five females who had previously received SOC and several rescue therapies.

Five of six patients had a clinical response at 6 months and reached a 12-month observation period. All five showed a significant reduction of proteinuria, an improvement of renal function and an increase of complement component levels, especially C4. The evaluation of SLEDAI-2K profiles confirmed the clinical effectiveness of the treatment in these patients. Regarding other non-conventional biomarkers, the decrease of INF-γ levels was especially relevant. Due to the influence of endogenous IFN inducers^24^, the activation of diverse IFN-producing cells^25^ and a genetic setup favoring IFN production^26^, SLE is characterized by an activation of the IFN system with an increased expression of IFN-regulated genes that promote a continuous stimulation of the immune system^27^. Our data suggest that daratumumab can interrupt the ongoing production of IFN-γ that sustains the autoimmune process in SLE. Receptor sBCMA, which is known to be increased in SLE (together with its ligands sAPRIL and sBAFF) also markedly dropped under daratumumab. Receptor sBCMA is strongly related to disease activity^28^. A significant increase in IL-10 levels was detected after daratumumab. IL-10 has pleiotropic effects in immunoregulation and inflammation. It downregulates the expression of Th1 cytokines and MCHC class II antigens, blocks NF-kB activity, regulates the JAK-STAT signaling and decreases the expression of co-stimulatory molecules on macrophages^29^. Our data also showed a significant decrease in sCD163 after daratumumab treatment. Soluble CD163 results from the enzymatic release of monocyte and macrophage cell membrane CD163 under inflammatory conditions^30^. Notably, sCD163, IFN-γ, IL-10 and BCMA were correlated with each other, and, most importantly, SLEDAI-2K values were directly correlated with IFN-γ, sBCMA and sCD163 levels and inversely correlated with IL-10.

Anti-DNA antibodies showed a relative delay in dropping under daratumumab, and a transitory increase in anti-DNA levels could be detected in coincidence with a SARS-CoV-2 infection and vaccination. Anti-DNA antibodies are mainly produced by B lymphocytes, and short-lived PCs, which are targeted by anti-CD20 monoclonal antibodies, are strongly influenced by anti-BLISS monoclonal antibodies but only indirectly influenced by anti-CD38 agents. That possibly explains the susceptibility of patients under daratumumab treatment to develop anti-DNA antibodies after strong immunological triggers, such as SARS-CoV-2 infection or vaccination. Nevertheless, abolishing the pleiotropic action of CD38 together with the decrease of IFN-γ production, sBCMA and sCD163 dropping and IL-10 increase likely provide for a rebalancing of the immune system and stabilization of clinical manifestations despite the latency of anti-DNA disappearance.

Our study has several limitations. First, albeit in our experience daratumumab was well tolerated, long-term data are needed to confirm the overall safety profile in the setting of SLE. Nevertheless, despite some intrinsic differences also due to the concomitant therapies used, our observation is in line with the overall good tolerability observed in the hematology/oncology use^31,32^. Second, our results need to be confirmed in adequately designed trials to reduce the potential bias related to patient selection, impact of previous and ongoing treatments (including intravenous steroids given concomitant to daratumumb as premedication) and the non-randomized design. In addition, future studies should consider a larger set of immunological parameters (at both serological and cellular levels) to improve understanding of the daratumumab-related impact on the immune system. Currently, a mono-center, open-label study to evaluate the safety and efficacy of daratumumab in combination with standard background therapy in participants with moderate to severe SLE (DARALUP, ClinicalTrials.gov identifier: NCT04810754) is ongoing. The therapeutic targeting of CD38 is anticipated to have impacts on different levels of immune response because of its expression and function in several immune cells. Among others, natural killer (NK) cells primarily express CD38 in a constitutive way^32^; hence, it is presently debatable how inhibiting CD38 would affect NK cells. Research into PC dyscrasias revealed that the processes set off by anti-CD38 monoclonal antibodies ultimately cause the immune system to be activated against myeloma cell growth^33^. Further research is necessary to examine how daratumumab affects NK cells in the specific setting of SLE.

Daratumumab proved to be well tolerated and safe in the patients treated in this study. Two patients who had SARS-CoV-2 infections during the treatment recovered without sequelae. That was not surprising because daratumumab does not affect B cells, which are the main defenders against newly emerging infections. Finally, one patient, who completed the entire course of daratumumab (that is, 24 infusions) and has been further followed for 6 months after daratumumab discontinuation without receiving any further immunosuppressant regimen, still presents with stable renal function and a moderate proteinuria, which may be the expression of residual fibrotic sequelae.

In summary, daratumumab could be a new effective therapeutic tool for the management of refractory LN and warrants further study.

## Methods

The entire series of patients comprises six adults with refractory LN who received daratumumab monotherapy. The results refer to the five patients reaching a 12-month follow-up from starting therapy and one patient who discontinued the treatment due to the absence of clinical response at month 6. All patients fulfilled the American College of Rheumatology classification criteria for SLE and had severe biopsy-proven renal involvement. Renal biopsy classification was performed as described in the 2003 International Society of Nephrology/Renal Pathology Society classification^34^. First-line treatment with SOC (MMF and CYC/azathioprine) failed in these patients. Additionally, as detailed in Table 1, previous treatments with rescue therapies (including RTX, belimumab, ocrelizumab and intravenous IgG) did not induce a clinical response. Patients who were given anti-B cell treatments (including CYC) were declared to be resistant after ≥6 months of observation after the therapeutic regimen.

**Table 1:** Patients treated with daratumumab reaching a 12-month follow-up

	Sex/gender^a^	Age at the onset (years)	Disease duration (years)	Previous treatments	LN class^b^ (A/C)^c^	Cumulative prednisone dose during the study (mg)	Residual steroid dose at 12 months^d^
Pt 1	M/M	41	9	GC,RTX+CYC,Bel,CYC,AZA	IV+V (7/4)	2,245	Prednisone 5 mg per day (13 months since RB)
Pt 2	F/W	24	5	GC,MMF,RTX + CYC	V (6/5)	3,012	Prednisone 7.5 mg per day (14 months since RB)
Pt 3	F/W	61	8	GC,RTX,CYC (high doses)	IV+V (8/5)	2,855	Prednisone 5 mg per day (13 months since RB)
Pt 4	F/W	20	10	MMF,RTX,CyA,Bel	III+V (7/4)	1,925	Prednisone 5 mg per day (14 months since RB)
Pt 5	F/W	54	20	GC,MMF,IgG iv,Bel	IV (7/4)	2,345	Prednisone 5 mg per day (13 months since RB)

Bel, belimumab; iv, intravenous; AZA, azathioprine, RTX, rituximab, MMF, mycophenolate mofetil, GC, glucocorticoids, CYC, cyclophosphamide, CyA, cyclosporine.

^a^Sex (male/female) and gender (man/woman) of participants was determined based on self-report.

^b^All patients underwent a kidney biopsy within 2 months before starting daratumumab.

^c^Activity (A) and chronicity (C) scores.

^d^Time (months) since renal biopsy in brackets. RB, renal biopsy.

The planned therapeutic protocol of daratumumab monotherapy consisted of 16 mg kg^−1^ weekly intravenous administrations for eight consecutive weeks and then every 2 weeks for eight more times and then monthly for another eight times. Premedication included paracetamol 1,000 mg (oral), chlorphenamine 10 mg (intravenous) and methylprednisolone 125 mg (intravenous).

Renal response was stratified as (1) complete renal response: proteinuria <0.5 g per 24 h, normal or near-normal eGFR (within 10% of normal eGFR if previously abnormal); (2) partial renal response: ≥50% reduction in proteinuria to sub-nephrotic levels (<3.5 g per 24 h) and normal or near-normal eGFR; and (3) no renal response: all the other cases^34^. The SLEDAI-2K index was calculated at each appointed visit as previously described^35^. Sex and/or gender of participants was determined based on self-report.

This study was conducted according to the Piedmont and Aosta Valley (Northwest Italy) legislation for Rare Diseases (N. 1577/UC/SAN of 11.10.2005 based on Regional Government Act 23 April 2007 dealing with Rare Diseases; article. 1: 796 paragraph Z Law Number 296 of 2006. Number 5-5740). Ethical Committee approval number 00323/2020 (Il Comitato Etico Interaziendale A.O.U. Città della Salute e della Scienza di Torino - A.O. Ordine Mauriziano - A.S.L. Città di Torino). The study was conducted according to the Declaration of Helsinki, and each patient provided written consent to participate. Patients were not treated as a part of a trial.

### Cytokine and soluble receptor serum testing

The serum of enrolled patients was collected at enrollment and during the follow-up and stored at −20 °C. Cycles of repeated freezing and thawing were avoided. sBMCA levels (detection limits 10 pg ml^−1^) were assessed by ELISA (R&D Systems). The serum levels of soluble CD163, IL-10 and IFN-γ were tested by a Bio-Plex Pro Human Cytokine Panel kit (Bio-Rad) according to the manufacturer’s protocols.

### Statistical analysis

Normality of data distributions was assessed by the Shapiro–Wilk test. Categorical variables are shown as absolute values and percentages, and continuous variables are reported as medians and minimum–maximum range. The Spearman rank correlation coefficient was used to assess correlations between parameters. The Wilcoxon matched‐pairs signed‐rank test was applied to investigate variations in parameters after treatment. Data were collected in Excel (Microsoft Office version 16.74). Data analysis was completed using SPSS version 28 (IBM Corporation) and GraphPad Prism 8.0 (GraphPad Software). *P* ≤ 0.05 was considered significant.

### Reporting summary

Further information on research design is available in the Nature Portfolio Reporting Summary linked to this article.

## Online content

Any methods, additional references, Nature Portfolio reporting summaries, source data, extended data, supplementary information, acknowledgements, peer review information; details of author contributions and competing interests; and statements of data and code availability are available at 10.1038/s41591-023-02479-1.

## Supplementary information

Supplementary Information

Reporting Summary

## Data Availability

Numerical data are detailed in Supplementary Table 2. All other data are included in the paper and supplementary documents. Correspondence and requests for materials or data should be addressed to Dario Roccatello, University of Torino. Requests will be processed in 10 working days.
