# Supplementary material for: Daratumumab monotherapy for refractory lupus nephritis
Source: Nat Med. 2023 Aug 10;29(8):2041–7. doi: 10.1038/s41591-023-02479-1 (PMC10427415; doi:10.1038/s41591-023-02479-1)
Supplement: Supplementary file 1 — Supplementary Information [file 41591_2023_2479_MOESM1_ESM.pdf]

nature medicine

Article https://doi.org/10.1038/s41591-023-02479-1

Daratumumab monotherapy for refractory lupus nephritis

In the format provided by the authors and unedited

Table 1S: Clinical and laboratory paraments before daratumumab. Discontinuation of previous agents occurs at least 6 months before starting daratumumab for B-cell depleting agents, including cyclophosphamide. CYC: cyclophosphamide, AZA: azathioprine, RTX: rituximab, Bel: belimumab, Ocr: ocrelizumab.

	Last regimen before daratumumab	sCr (mg/dl)	Proteinuria g/24 h	SLEDAI-2K
Pt 1	CYC+AZA	2.1	9	14
Pt 2	RTX+CYC	0.6	8.5	8
Pt 3	CYC	1.1	6	10
Pt 4	Bel	0.6	2	8
Pt 5	Bel	7.0	3	14
Pt 6	Ocr	0.6	7	10

Table 2S: Laboratory profiles of the 6 patients.

Patients	Hb; g/dl (time 0, 3, 6, 9 and 12 months) [12.5-16]	WBC (time 0, 3, 6, 9 and 12 months) [10*3/microl]	sCr (mg/dl) (time 0, 3, 6, 9 and 12 months) [0.5-0,96]	C3 (mg/dl) (time 0, 3, 6, 9 and 12 months) [90-180]	C4 /mg/dl) (time 0, 3, 6, 9 and 12 months) [10-40]	anti-dsDNA (UI/mL) (time 0, 3, 6, 9 and 12 months) [<15]	CD19/20+ B cell numbers (time 0, 3, 6, 9 and 12 months) [cell/microl]	Hematuria (time 0, 3, 6, 9 and 12 months)	uPT g/24h (time 0, at 3, 6, 9 and 12 months) [<0.3]	CrCl (ml/min) (time 0, at 3, 6, 9 and 12 months) [85-125]
Pt 1	14.9/13.3/15.0/15.2	7230/7760/6790/6760	2.1/1.3/1.3/1.5	58/112/89/80	12/25/18/19	378/124/12/12	41/28/15/28	++/+-/+/+	9.8/1.5/1.2/2.0	52/90/90/81
Pt 2	12.1/11.1/12.1/10.9	6470/5510/7210/5400	0.6/0.6/0.6/0.6	93/146/60/82	14/16/6/13	127/43/12/12	565/375/610/244	++/+/-/-	8.0/0.1/2.6/1.5	162/162/162/162
Pt 3	11.1/12.1/10.9/10.5	5510/7210/5290/5460	1.2/1.6/1.3/1.3	20/74/81/96	5/22/22/22	157/65/77/12	60/44/42/35	++/+-/+-/+-	5.5/0.3/0.3/0.1	42/31/38/38
Pt 4	13.1/12.9/12.3/12.3	9160/9310/7160/8240	0.6/0.7/0.6/0.6	94/109/99/115	10/19/18/18	321/12/12/12	76/62/58/91	-/-/-/-	2/0.4/0.4/0.3	145/124/145/145
Pt 5	8.6/9.0/9.1/10.6	3850/4560/6140/7950	7.0/3.5/3.6/3.3/	99/132/139/135	6/26/28/30	12/12/121/154	98/90/78/50	++/-/-/-	2.8/0.7/0.1/0.1	11/22/21/23
Pt 6	12.2/12.7/12.3/11.4	3560/10430/4510/4410	0.6/0.6/0.6/0.7	76/85/144/140	7/12/12/12	15/12/12/15	109/118/154/134	++/++/++/++	7.2/6/5.5/6.0	109/109/109/94
