# Supplementary material for: Daratumumab monotherapy for refractory lupus nephritis
Source: Nat Med. 2023 Aug 10;29(8):2041–7. doi: 10.1038/s41591-023-02479-1 (PMC10427415; doi:10.1038/s41591-023-02479-1)
Supplement: Supplementary file 2 — Reporting Summary [file 41591_2023_2479_MOESM2_ESM.pdf]

nature portfolio

Corresponding author(s): Dario Roccatello

Last updated by author(s): Jun 26, 2023

Reporting Summary

Nature Portfolio wishes to improve the reproducibility of the work that we publish. This form provides structure for consistency and transparency in reporting. For further information on Nature Portfolio policies, see our Editorial Policies and the Editorial Policy Checklist.

Statistics

For all statistical analyses, confirm that the following items are present in the figure legend, table legend, main text, or Methods section.

n/a	Confirmed	
☐	☒	The exact sample size (*n*) for each experimental group/condition, given as a discrete number and unit of measurement
☐	☒	A statement on whether measurements were taken from distinct samples or whether the same sample was measured repeatedly
☐	☒	The statistical test(s) used AND whether they are one- or two-sided<br>*Only common tests should be described solely by name; describe more complex techniques in the Methods section.*
☒	☐	A description of all covariates tested
☒	☐	A description of any assumptions or corrections, such as tests of normality and adjustment for multiple comparisons
☐	☒	A full description of the statistical parameters including central tendency (e.g. means) or other basic estimates (e.g. regression coefficient) AND variation (e.g. standard deviation) or associated estimates of uncertainty (e.g. confidence intervals)
☐	☒	For null hypothesis testing, the test statistic (e.g. *F*, *t*, *r*) with confidence intervals, effect sizes, degrees of freedom and *P* value noted<br>*Give P values as exact values whenever suitable.*
☒	☐	For Bayesian analysis, information on the choice of priors and Markov chain Monte Carlo settings
☒	☐	For hierarchical and complex designs, identification of the appropriate level for tests and full reporting of outcomes
☐	☒	Estimates of effect sizes (e.g. Cohen's *d*, Pearson's *r*), indicating how they were calculated

Our web collection on statistics for biologists contains articles on many of the points above.

Software and code

Policy information about availability of computer code

Data collection	Data Were collected in Excel, Microsoft Office version 16.74
Data analysis	Data analysis was completed using SPSS v.28 (IBM Corporation; Armonk, NY, USA) and GraphPad Prism 8.0 (GraphPad Software Incorporation; La Jolla, CA, USA). A p-value ≤ 0.05 was considered significant.

For manuscripts utilizing custom algorithms or software that are central to the research but not yet described in published literature, software must be made available to editors and reviewers. We strongly encourage code deposition in a community repository (e.g. GitHub). See the Nature Portfolio guidelines for submitting code & software for further information.

Data

Policy information about availability of data

All manuscripts must include a data availability statement. This statement should provide the following information, where applicable:

- Accession codes, unique identifiers, or web links for publicly available datasets
- A description of any restrictions on data availability
- For clinical datasets or third party data, please ensure that the statement adheres to our policy

Numerical data are detailed in Table 2S. Any additional data will be provided after reasonable to the corresponding author.

Human research participants

Policy information about studies involving human research participants and Sex and Gender in Research.

Reporting on sex and gender	Sex and/or gender of participants was determined based on self-report.
Population characteristics	Due to the limited sample size and the clinical nature of our study, only a limited set of variables where computed, and they were collected in line with the clinical practice. Variables are detailed in a not-aggregated form in Table 1
Recruitment	Patients were identified among those patients attending the center who were refractory to previous treatment. All patients who met the inclusion criteria (No response to the first line treatment with SOC (MMF, CYC/azathioprine) were recruited.
Ethics oversight	Daratumumab was obtained by the local hospital pharmacy for off-label use, according to the rules for the management of Rare Diseases of Piedmont (North-West Italy). All patients provided informed consent. No commercial sponsor was involved.

Note that full information on the approval of the study protocol must also be provided in the manuscript.

Field-specific reporting

Please select the one below that is the best fit for your research. If you are not sure, read the appropriate sections before making your selection.

☒ Life sciences ☐ Behavioural & social sciences ☐ Ecological, evolutionary & environmental sciences

For a reference copy of the document with all sections, see nature.com/documents/nr-reporting-summary-flat.pdf

Life sciences study design

All studies must disclose on these points even when the disclosure is negative.

Sample size	Our study is a case-series. No sample size was calculated. For the laboratory parameters, N=5 biologically independent samples were used for each determination (1 per patient/time point).
Data exclusions	No data were excludeed.
Replication	For the laboratory parameters, N=5 biologically independent samples were used for each determination (1 per patient/time point).
Randomization	Our study is a case-series. Study designed did not include randomization.
Blinding	Treatment was given open in a single arm study. No blinding was planned.

Reporting for specific materials, systems and methods

We require information from authors about some types of materials, experimental systems and methods used in many studies. Here, indicate whether each material, system or method listed is relevant to your study. If you are not sure if a list item applies to your research, read the appropriate section before selecting a response.

Materials & experimental systems

n/a	Involved in the study	
☐	☒	Antibodies
☒	☐	Eukaryotic cell lines
☒	☐	Palaeontology and archaeology
☒	☐	Animals and other organisms
☐	☒	Clinical data
☒	☐	Dual use research of concern

Methods

n/a	Involved in the study	
☒	☐	ChIP-seq
☒	☐	Flow cytometry
☒	☐	MRI-based neuroimaging

Antibodies

Antibodies used	Daratumumab as commercially available for therapeutic use
Validation	N/A

Clinical data

Policy information about clinical studies

All manuscripts should comply with the ICMJE guidelines for publication of clinical research and a completed CONSORT checklist must be included with all submissions.

Clinical trial registration	N/A
Study protocol	Treatment protocol of Daratumumab monotherapy consisted of 16 mg/kg weekly intravenous administrations for 8 consecutive weeks, then every two weeks for 8 more times, and lastly monthly for a maximum of 24t administrations. Premedication included paracetamol 1000 mg (oral), chlorphenamine 10 mg (intravenous), and methylprednisolone 125 mg (intravenous).
Data collection	The entire series of patients comprises 6 adults with refractory lupus nephritis who received Daratumumab monotherapy. The results refer to the 5 patients reaching a 12-month follow-up from starting therapy and 1 patient who discontinued the treatment due to the absence of clinical response at month 6th
Outcomes	Renal response was stratified as: (i): complete renal response: proteinuria <0.5 g/24 hours, normal or near-normal estimated glomerular filtration rate (within 10% of normal estimated glomerular filtration rate if previously abnormal); (ii): partial renal response: ≥50% reduction in proteinuria to sub-nephrotic levels (<3.5 g/24 hours), and normal or near-normal estimated glomerular filtration rate; (iii) no renal response: all the other cases
